# Supplementary material for: Complexome profiling of the Chlamydomonas psb28 mutant reveals TEF5 as an early PSII assembly factor
Source: Plant Cell. 2025 Mar 17;37(6):koaf055. doi: 10.1093/plcell/koaf055 (PMC12164590; doi:10.1093/plcell/koaf055)
Supplement: koaf055_Supplementary_Data [file koaf055_supplementary_data.zip › psb28_interactive.zip!/plots/intensity/qualityPlots/peptides/CEP1_Cre09.g407700.t1.2.html]

### Cre09.g407700.t1.2

Trivial name: CEP1  
SeqCoverage [median]: 11.40  
Mass: 53.92 kDa  
  
 

| Sequence | MissedCleavage | Mass [Da] | wtIntensity | muIntensity | Score |
| --- | --- | --- | --- | --- | --- |
| AAAHQPVAVAIEADAK | 0 | 1560.83 | 9309.11 | 8978.75 | 80.10 |
| NAVAEVK | 0 | 729.40 | 1068.91 | 1760.13 | 87.32 |
| RVVTIDSYEDVPENDEVALKK | 2 | 2418.23 | 0.00 | 1469.27 | 61.28 |
| VVTIDSYEDVPENDEVALKK | 1 | 2262.13 | 629.64 | 10291.64 | 83.42 |
|  |  |  | 11007.7 | 22499.8 |  |
